# Advanced Maternal Age Worsens Postpartum Vascular Function

**DOI:** 10.3389/fphys.2017.00465

**Published:** 2017-06-30

**Authors:** Jude S. Morton, Alison S. Care, Raven Kirschenman, Christy-Lynn Cooke, Sandra T. Davidge

**Affiliations:** ^1^Department of Obstetrics and Gynaecology, University of AlbertaEdmonton, AB, Canada; ^2^Women and Children's Health Research InstituteEdmonton, AB, Canada; ^3^Department of Physiology, University of AlbertaEdmonton, AB, Canada

**Keywords:** advanced maternal age, postpartum, vascular function, prostaglandin, pregnancy loss

## Abstract

The age at which women experience their first pregnancy has increased throughout the decades. Pregnancy has an important influence on maternal short- and long-term cardiovascular outcomes. Pregnancy at an advanced maternal age increases maternal risk of gestational diabetes, preeclampsia, placenta previa and caesarian delivery; complications which predict worsened cardiovascular health in later years. Aging also independently increases the risk of cardiovascular disease; therefore, combined risk in women of advanced maternal age may lead to detrimental cardiovascular outcomes later in life. We hypothesized that pregnancy at an advanced maternal age would lead to postpartum vascular dysfunction. We used a reproductively aged rat model to investigate vascular function in never pregnant (virgin), previously pregnant (postpartum) and previously mated but never delivered (nulliparous) rats at approximately 13.5 months of age (3 months postpartum or equivalent). Nulliparous rats, in which pregnancy was spontaneously lost, demonstrated significantly reduced aortic relaxation responses (methylcholine [MCh] Emax: 54.2 ± 12.6%) vs. virgin and postpartum rats (MCh Emax: 84.8 ± 3.5% and 84.7 ± 3.2% respectively); suggesting pregnancy loss causes a worsened vascular pathology. Oxidized LDL reduced relaxation to MCh in aorta from virgin and postpartum, but not nulliparous rats, with an increased contribution of the LOX-1 receptor in the postpartum group. Further, in mesenteric arteries from postpartum rats, endothelium-derived hyperpolarization (EDH)-mediated vasodilation was reduced and a constrictive prostaglandin effect was apparent. In conclusion, aged postpartum rats exhibited vascular dysfunction, while rats which had pregnancy loss demonstrated a distinct vascular pathology. These data demonstrate mechanisms which may lead to worsened outcomes at an advanced maternal age; including early pregnancy loss and later life cardiovascular dysfunction.

## Introduction

Pregnancy has been considered as a “window” to future cardiovascular health, influencing both short- and long-term cardiovascular outcomes for both the mother and the child (Jacobs et al., 2012; Cain et al., 2016). At a younger age, pregnancy is considered to be cardioprotective when compared to nulligravidity (Clapp Iii and Capeless Md, 1997; Jacobs et al., 2012). However, the age at which women first become pregnant has gradually increased throughout the decades. This change has been driven by many factors such as relationship status, finances and education (Proudfoot et al., 2009). In Western societies such as the US, the number of women who gave birth to their first child when they were over the age of 35 increased from 5.8% in 1994 (1996) to 9.1% in 2014 (Martin et al., 2009). Similarly, in Canada first births to women over 35 accounted for 11.2% of all births in 2009 (2012).

Pregnancy at an advanced maternal age increases the prevalence of maternal morbidity including gestational diabetes, preeclampsia, placenta previa and caesarian delivery (van Katwijk and Peeters, 1998; Jacobsson et al., 2004; Cleary-Goldman et al., 2005; Pawde et al., 2015). The occurrence of pregnancy complications such as gestational diabetes, preeclampsia, low birth weight infants, and preterm delivery, independently predicts worsened cardiovascular health in women in later years (Catov et al., 2007, 2008; Gongora and Wenger, 2015; Leslie and Briggs, 2016). Further, aging itself, without the added stressor of pregnancy, is known to increase the risk of cardiovascular disease in the general population (Merz and Cheng, 2016; Paolo Emilio et al., 2016). It is likely, therefore, that these risks may accumulate in women of advanced maternal age and cause detrimental cardiovascular outcomes in later life.

Despite the steadily increasing prevalence of women conceiving later in life, few epidemiological data exist which define the risk for these women in postpartum life and the data which does exist is contradictory. A study reporting on advanced maternal age pregnancies (35+ years) in a total cohort of 6,559 women showed that there was a risk of increased systolic blood pressure, poor health assessment by a physician and poor mobility in women. In their analysis, however, 130 advanced maternal age first births were grouped together with 1,294 women whose first birth was not after 35 years of age; thereby masking the potentially greater detrimental effects of first births at an advanced maternal age (Alonzo, 2002). The distinction of first vs. subsequent pregnancies is an important one since a higher gravidity has been associated with cardioprotective mechanisms (Jacobs et al., 2012). In terms of gravidity, however, a higher incidence of cardiovascular disease has also been demonstrated in postpartum women with no pregnancy complications compared to nulliparous women (Catov et al., 2008); demonstrating the perplexing nature of this field. Further complicating the determination of cardiovascular risk in women of advanced maternal age, one study has shown a decreased likelihood of high blood pressure in later life with increased age at first birth (Lind et al., 2015), while another demonstrated no increase in cardiovascular risk with maternal age (Huisman et al., 2013). The latter study, however, was underpowered with only an n of 26 advanced maternal age pregnancies in a total of 84.

A common early pathology of many detrimental cardiovascular outcomes is vascular dysfunction. For instance, aging is associated with increased vascular stiffness and endothelial dysfunction (reviewed in Moreau and Hildreth, 2014; Camici et al., 2015), both of which contribute to the development of hypertension (reviewed in Faconti et al., 2015; Harvey et al., 2015). Further, many obstetrical complications are associated with vascular dysfunction (reviewed in Enkhmaa et al., 2016). Indeed, decreased endothelium-dependent vasodilation/increased vasoconstriction, decreased sensitivity to nitric oxide (NO)/increased sensitivity to angiotensin II (AngII) and endothelin-1 (ET-1), decreased antioxidant capacity/increased oxidative stress, increased arterial stiffness and inflammatory factors are all common to both aging and complications of pregnancy (Morton and Davidge, 2013; Gongora and Wenger, 2015; Barton et al., 2016; Collins et al., 2016; Wray et al., 2017). Few studies have been performed, however, to investigate whether these factors could also be responsible for increased cardiovascular risk following a pregnancy that is complicated by advanced maternal age. A previous study in our laboratory has shown that advanced maternal age in the rat resulted in reduced fertility, reduced litter size, increased maternal systolic blood pressure and altered vascular function in the peripartum period (Care et al., 2015). These data are in line with human epidemiological data which demonstrate an increased incidence of both adverse acute maternal (hypertension, diabetes, miscarriage, caesarian section) and neonatal (intrauterine growth restriction, low birth weight infants, malformations, low Apgar scores, perinatal mortality) outcomes in aging mothers (Cleary-Goldman et al., 2005; Salem Yaniv et al., 2011; Almeida et al., 2015; Schimmel et al., 2015; Traisrisilp and Tongsong, 2015). To investigate the mechanisms of detrimental cardiovascular outcomes following pregnancy at an advanced maternal age, we used a rat model of advanced maternal age to assess maternal vascular function at 3 months postpartum. We hypothesized that advanced maternal age in rats would lead to increased vascular dysfunction in the postpartum period compared to age-matched virgin controls.

## Materials and methods

### Ethical approval

All experimental protocols were approved by the University of Alberta Health Sciences Animal Policy and Welfare Committee in accordance with the Canadian Council on Animal Care guidelines; AUP # 00000242.

### Animal model

Female Sprague Dawley rats were purchased from Charles River (St. Constant, QC) at 3 months of age, and housed in the Animal Care Facility at the University of Alberta. Rats were maintained on *ad libitum* access to standard rat chow and water in a 10:14 h light:dark cycle and an ambient temperature of 22 ± 1°C. At 9.5–10 months of age, rats in the aged pregnancy group (postpartum) were mated overnight with 3–5 month old males and gestational day (GD) 0 was confirmed by the presence of sperm in a vaginal smear. In the rat, 9.5–10 months of age corresponds to approximately 35 years of age in humans (i.e., defined for humans as advanced maternal age), when considering milestones such as weaning, sexual maturity, skeletal maturity and reproductive senescence (Sengupta, 2011). Litters remained with their dams until weaning at 3 weeks of age; at which point the offspring were kept for a separate study. The control group consisted of similarly aged rats which had never been pregnant (virgin). Rats from each of the experimental groups were further aged until experimental use at 3 months postpartum (or equivalent age in virgin rats); corresponding to approximately 52 years of age, or 17 years postpartum, in humans. Hence, the primary study groups consisted of approximately 13.5 month old rats in the following designations: aged never pregnant (virgin, *n* = 5; body weight 521 ± 63 g) and aged delivered (postpartum, *n* = 12; body weight 469 ± 18 g).

As detailed in our previous publication, rats of an advanced maternal age have a significantly reduced viable pregnancy rate (Care et al., 2015). Similarly, in the current study some rats were tested positive for sperm but failed to deliver a litter at GD22 (nulliparous). Initially considered as a control group for the test subjects of advanced maternal age gestation, these animals which spontaneously lost their litter were later separated and considered, at the equivalent of 3 months postpartum (i.e., 3 months after their anticipated delivery date had they remained pregnant, ~13.5 months old), as a separate experimental group based on their vascular outcomes: aged no delivery (nulliparous, *n* = 5; body weight 545 ± 68 g). The timing of pregnancy loss in aged pregnancies (9.5–10 months old) was further investigated in a separate cohort of animals which were euthanized at GD5 (*n* = 6) or GD8 (*n* = 11). In this cohort, pregnancy outcomes were compared to young (3–4 month old) pregnant controls (*n* = 17).

### Vascular function assessments

Vascular function was assessed in aorta and mesenteric resistance arteries from 3 month postpartum rats and their respective controls. Aortae were assessed as a reference conduit artery in which expression of scavenger receptors has been shown to be increased in complicated pregnancies such as preeclampsia (Morton et al., 2012), and in models of diabetes (Chen et al., 2001), hypertension (Nagase et al., 1997), and atherosclerosis (Chen et al., 2000; Fisker Hag et al., 2008; Tsoukatos et al., 2008). Mesenteric arteries were assessed due to their role as a resistance artery important in the control of systemic blood pressure (Greenway, 1983; Rothe, 1983). Animals were euthanized via inhaled isoflurane (4% in air) followed by exsanguination. A short, 30 min, duration of exposure to isoflurane has been shown not to induce reactive oxygen species production in cultured cells (Sun et al., 2014) and, therefore, is considered to be an appropriate method of euthanasia. The thoracic aorta and 2nd order mesenteric arteries were rapidly excised and cleaned of surrounding connective tissues in ice cold, HEPES-buffered physiological saline solution (PSS; in mmol/L: 142 NaCl, 4.7 KCl, 1.17 MgSO_4_, 4.7 CaCl_2_, 1.18 K_2_PO_4_, 10 HEPES, 5.5 glucose, pH 7.4). Arteries were mounted on an isometric myograph system (DMT, Copenhagen, Denmark), using 40 μm tungsten wire. Arteries were normalized through a series of stepwise increases in diameter to determine the 0.8_*L*100_ (mesenteric arteries) or adjusted to 1.5 g tension (aorta).

Following an equilibration period, vessels were treated twice with phenylephrine (PE; 10 μmol/L) and once with methylcholine (MCh; 3 μmol/L) to assess functionality of the vascular smooth muscle and endothelium respectively. Aortic and mesenteric constriction was investigated using cumulative doses of PE (0.01–100 μmol/L). The influence of basal nitric oxide (NO) production on PE-induced constriction was tested by incubating arteries for 30 min with N^G^-nitro-L-arginine methyl ester (L-NAME; 100 μmol/L; a pan inhibitor of NO synthase).

Given the potential role of endothelial dysfunction, the primary pathways involved in endothelial modulation of vascular function were investigated. The involvement of NO, endothelium-dependent hyperpolarization (EDH) and prostaglandins (PG) in MCh-induced relaxation was assessed. Arteries were incubated for 30 min with inhibitors added to the bathing PSS medium prior to preconstriction with an EC_80_ concentration of PE and stimulation with cumulative doses of MCh (0.003–3 μmol/L). The influence of L-NAME (100 μmol/L); indomethacin (indo; 5 μmol/L; a non-selective COX-1 and -2 inhibitor), or a combination of Apamin (Apa; 100 nmol/L; a small-conductance, Ca^2+^-activated potassium channel (SK_Ca_) blocker) and 1-[(2-chlorophenyl) diphenylmethyl]-1H-pyrazole (TRAM-34; 10 μmol/L; an intermediate-conductance, Ca^2+^-activated potassium channel (IK_Ca_) blocker), on MCh-induced relaxation was tested.

Pregnancy at an advanced maternal age potentially combines the risks of aging and a pregnancy complication. Previous studies have shown that a scavenger receptor called the lectin-like oxidized lipoprotein-1 (LOX-1) receptor is upregulated in aging-associated diseases, such as atherosclerosis (Chen et al., 2000) and with pregnancy complications, such as preeclampsia (Morton et al., 2012). Therefore, to determine the involvement of scavenger receptors in advanced maternal age postpartum vascular function, responses to MCh were investigated in the presence or absence of oxidized low-density lipoprotein (oxLDL, 50 μg/mL, Kalen Biomedical; to stimulate scavenger receptors); antiLOX-1 antibody (TS20, 10 μg/mL; a LOX-1 receptor inhibitor generously donated by Tatsuya Sawamura); polyethylene glycol superoxide dismutase (pegSOD, 50 units/mL; a superoxide scavenger); or a combination of CV-6209 (CV, 0.01 μmol/L) and WEB 2086 (WEB, 0.5 μmol/L), inhibitors of the platelet activating factor (PAF) receptor.

At the end of the experiment, the bathing medium was changed to a 124 mmol/L potassium chloride solution (high KCl buffer; in mmol/L: 10 HEPES, 24 NaCl, 124 KCl, 2.4 MgSO_4_, 4.9 CaCl_2_, 1.18 KH_2_PO_4_, 5.5 glucose; pH 7.4) to assess maximal, non-receptor-mediated constriction.

### Implantation and resorption site assessments

On GD5, pregnant young or aged rats were anesthetized with isoflurane (5% induction, 2.5% maintenance, in air). The rat tail was cleaned with 70% ethanol and the tail vein was injected with 0.5 ml of trypan blue solution (Sigma; 0.4% diluted 1:1 with saline). After 10 min, rats were euthanized via cardiac puncture and uteri were dissected and assessed for clearly delineated blue bands as evidence of early implantation sites. In another subset of rats, uteri were extracted on GD8 and the number of healthy and resorbing implantation sites, visible to the naked eye at this stage of pregnancy, were counted.

### Statistical analyses

Data are presented as mean ± SEM and were plotted and analyzed using GraphPad Prism software version 6.0. For comparison of vascular function data, concentration-response curves were fitted to the Hill equation from which a sigmoid plot was generated by non-linear least squares regression analysis. The mean effective concentration that produced 50% of the maximal response (EC_50_) was then determined. Comparisons of summary E_max_, EC_50_, and area under curve (AUC) data were made using a Student's *t*-test, one-way ANOVA with Tukey's post-hoc test or two-way ANOVA with Sidak's *post-hoc* test for multiple comparisons, as required. The effect of maternal age on pregnancy viability was evaluated with the Fisher's Exact test. The number of implantation sites (GD5 and GD8) and embryo resorptions (GD8) in early pregnancy were analyzed by two-way ANOVA or *t*-test, as required. A *P* < 0.05 was considered statistically significant.

## Results

### Aortic vascular phenotype

**Figure 1:**
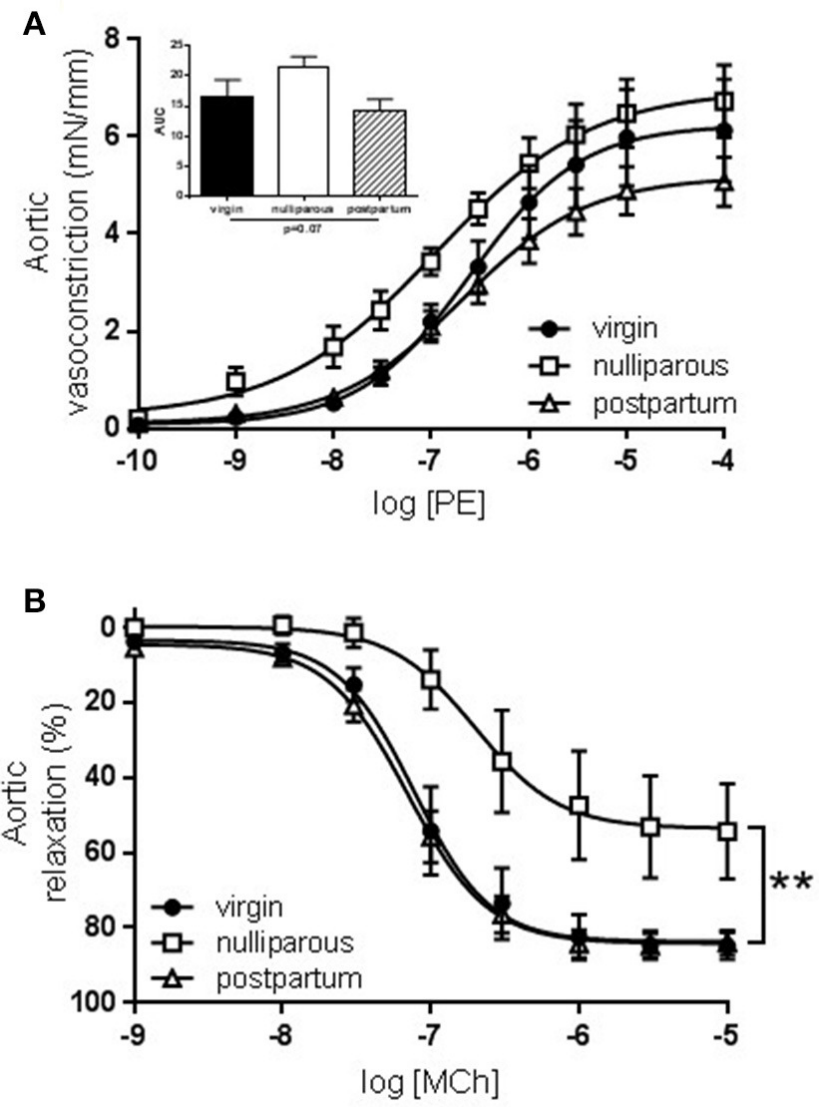
Constriction and relaxation responses of *aorta* from advanced maternal age virgin, nulliparous or postpartum rats at 3 months postpartum, or equivalent age in never pregnant controls (~13.5 months old). Constriction to **(A)** phenylephrine (PE) tended to be increased (*p* = 0.07) in aorta from nulliparous rats. Inset shows analysis of area under curve (AUC) summary data, *n* = 5–12; group effect *p* = 0.07; data analyzed by one-way ANOVA. **(B)** Relaxation to methylcholine (MCh) was significantly reduced (*p* = 0.007) in aorta from nulliparous rats compared to virgin and postpartum rats, *n* = 5–11. Data were analyzed by one-way ANOVA with Tukey's post-test: ^**^p < 0.01 nulliparous vs. postpartum and virgin groups.

**Figure 2:**
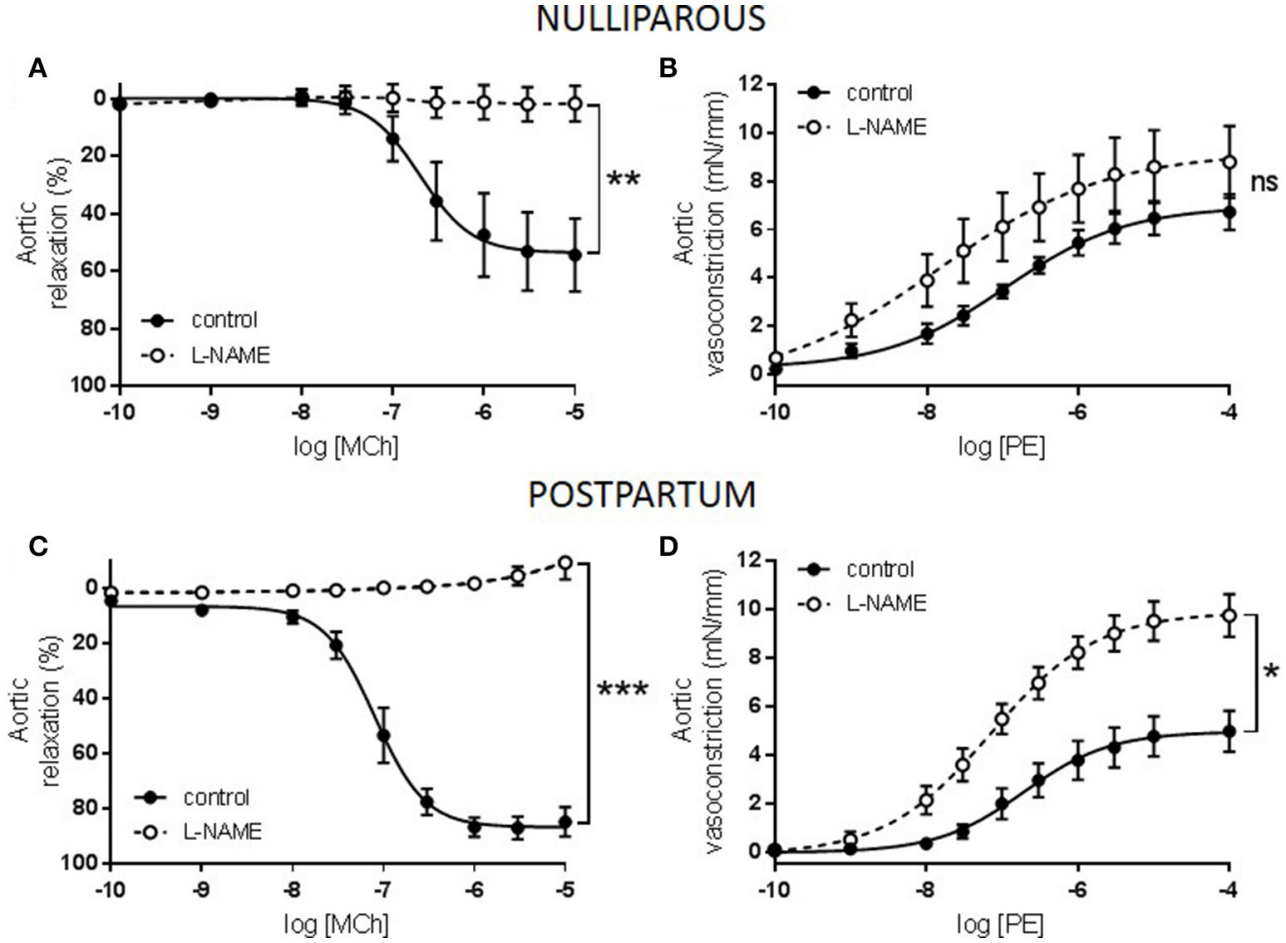
Effect of nitric oxide synthase inhibition on responses to methylcholine (MCh) and phenylephrine (PE) in *aorta* from advanced maternal age nulliparous or postpartum rats at 3 months postpartum (~13.5 months old). Relaxation to MCh was abolished in aorta from **(A)** nulliparous and **(C)** postpartum rats following inhibition of nitric oxide synthase (L-NAME) with a greater NO component in postpartum than nulliparous rats, *n* = 5/group. Constriction to PE was significantly increased following incubation with L-NAME in aorta from **(D)** postpartum but not **(B)** nulliparous rats, *n* = 5/group. The effect of parity and L-NAME on vasodilation to MCh (AUC) were analyzed by two-way ANOVA with Sidak's post-test: ^*^p < 0.05, ^**^p < 0.01, ^***^p < 0.001.

Constriction to KCl was unaltered between all three groups (data not shown) while responses to PE tended to be increased in only the nulliparous group (Figure 1A, *p* = 0.076). Similarly, maximal relaxation responses to MCh were significantly reduced in only the nulliparous group (Figure 1B, *p* = 0.007). Since the nulliparous group demonstrated the greatest reduction in MCh-induced relaxation, further investigation was performed into the potential mechanisms affected. The presence of L-NAME, a non-selective NOS inhibitor, completely abolished relaxation in both groups which carried offspring: the nulliparous and postpartum groups (Figures 2A,C). Analysis of the delta AUC demonstrated that there was a significant reduction in NO-mediated vasodilation in the nulliparous compared to the postpartum group (ΔAUC: nulliparous 91.4 ± 32.4 vs. postpartum 205.5 ± 19.7; *p* = 0.017). In addition, responses to PE were significantly increased by the inclusion of L-NAME in the postpartum group (ΔAUC: 17.4 ± 3.4) but not in the nulliparous group (ΔAUC: 11.9 ± 6.2) (Figures 2B,D); suggesting a reduction in basal NO production in animals in which pregnancy terminated early.

**Figure 3:**
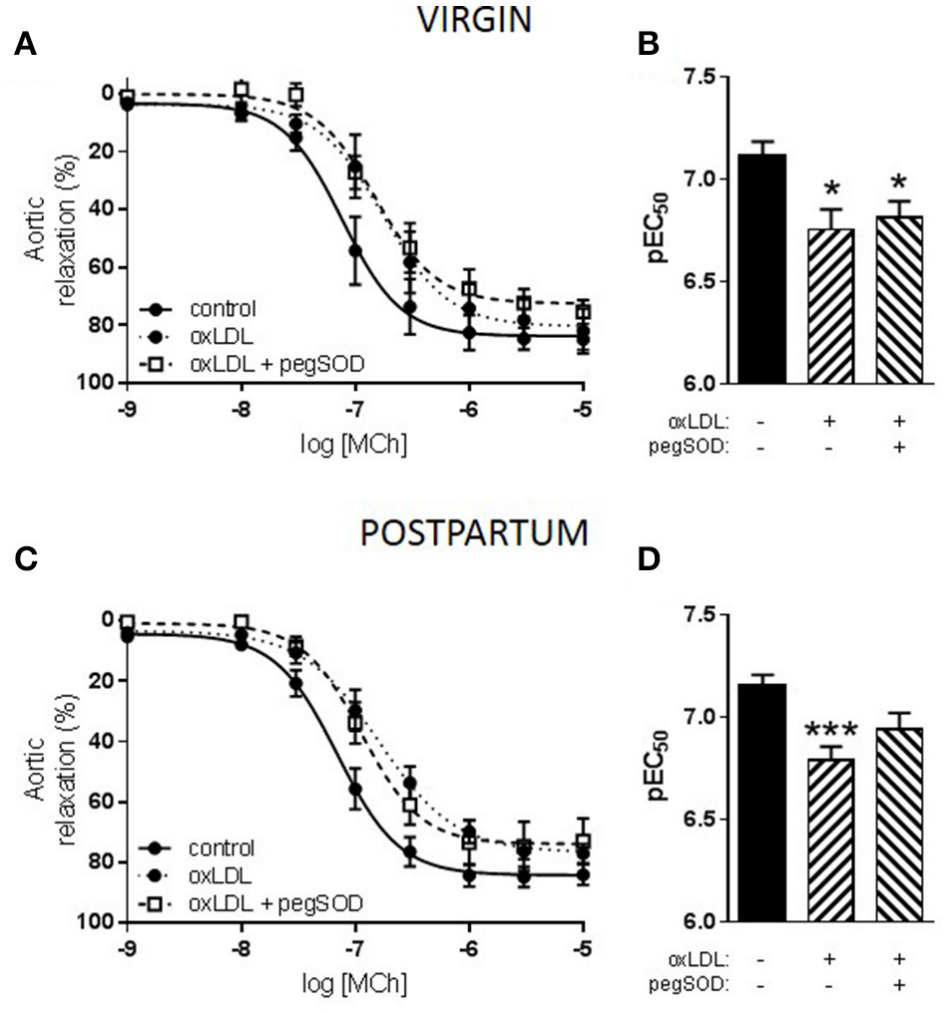
Effect of oxidized LDL (oxLDL) and superoxide scavenging on responses to methylcholine (MCh) in *aorta* from advanced maternal age postpartum rats at 3 months postpartum, or equivalent age in never pregnant (virgin) controls (~13.5 months old). Relaxation to MCh was significantly reduced by oxLDL in aorta from **(A)** virgin and **(C)** postpartum rats. Concurrent incubation with pegSOD, a superoxide scavenger, did not significantly prevent inhibition of MCh-induced vasodilation by oxLDL in either group. **(B,D)** Analysis of pEC_50_ summary data by one-way ANOVA with Sidak's post-test, ^*^p < 0.05, ^***^p < 0.001 vs. control; *n* = 4–11.

Stimulation of the LOX-1 receptor with oxLDL did not alter relaxation responses to MCh in the nulliparous group (pEC_50_: MCh 6.7 ± 0.2 vs. MCh + oxLDL 6.4 ± 0.2; ns). In both the virgin and postpartum groups, however, incubation with oxLDL significantly reduced sensitivity to MCh (Figure 3). Further investigation into the phenotype of the postpartum group compared to the virgin control was undertaken. One of the primary scavenger receptors which can be activated by oxLDL is the LOX-1 receptor; a downstream effect of which is increased production of superoxide anions. In both the virgin and postpartum groups, incubation with the superoxide scavenger pegSOD along with oxLDL did not prevent the reduction in relaxation that was seen to occur with oxLDL alone (Figures 3A–D).

**Figure 4:**
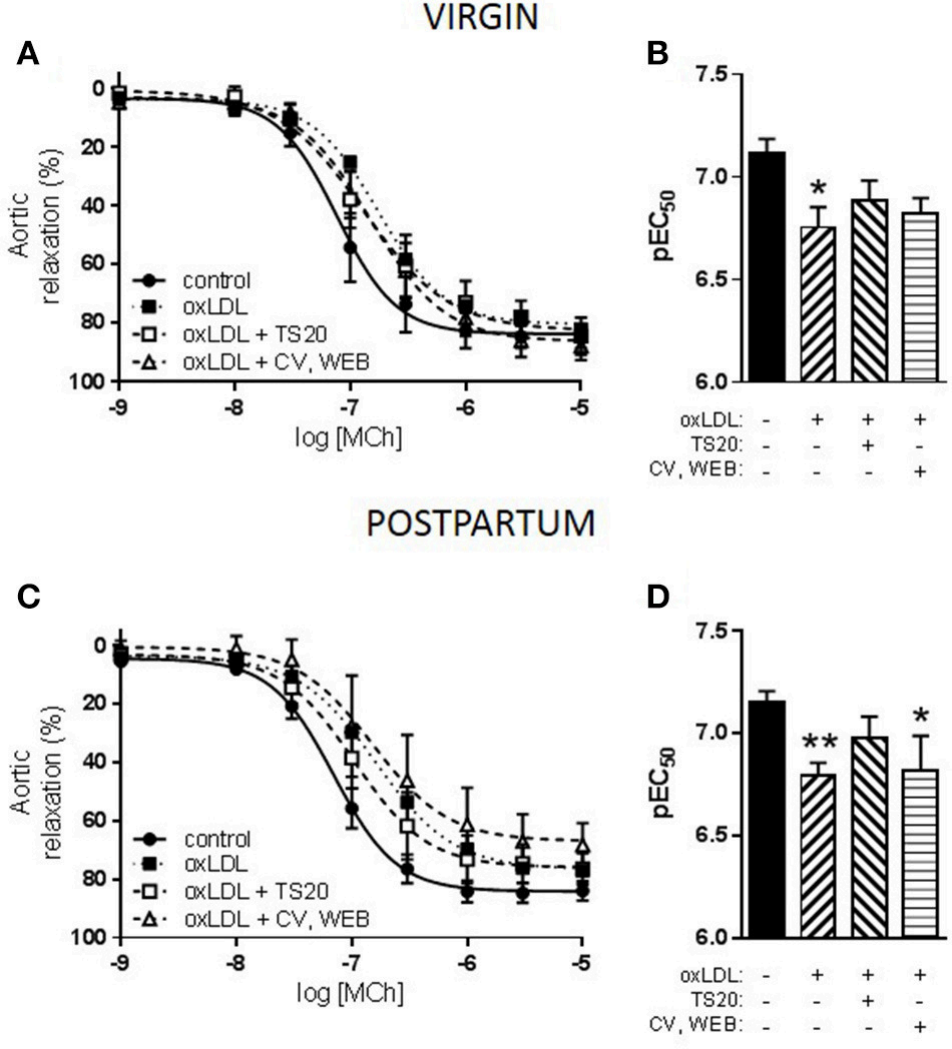
Effect of oxidized LDL (oxLDL) and scavenger receptor inhibitors on responses to methylcholine (MCh) in *aorta* from advanced maternal age postpartum rats at 3 months postpartum, or equivalent age in never pregnant (virgin) controls (~13.5 months old). **(A)** Concurrent incubation with either TS20 (LOX-1 inhibitor) or CV-6209 and WEB 2086 (CV, WEB; PAF inhibitors) did not prevent inhibition of MCh-induced vasodilation by oxLDL in virgin rats. **(C)** In postpartum rats, the presence of CV, WEB did not prevent oxLDL-induced dysfunction; however, in the presence of TS20 MCh-induced relaxation was partially recovered. **(B,D)** Analysis of pEC_50_ summary data by one-way ANOVA with Sidak's post-test, ^*^p < 0.05, ^**^p < 0.01 vs. control; *n* = 4–11.

The effect of inhibitors of the LOX-1 receptor (TS20), or the PAF receptor (CV and WEB) on oxLDL-induced dysfunction in both virgin and postpartum groups was also assessed. In the virgin group, neither TS20 nor CV/WEB treatments reversed the inhibitory effect of oxLDL on MCh-induced relaxation (Figures 4A,B). In the postpartum group, only the presence of the LOX-1 receptor inhibitor partially prevented the reduction of relaxation by oxLDL while there continued to be a significant reduction in MCh-induced relaxation in the presence of the PAF receptor inhibitors (Figures 4C,D).

### Mesenteric artery vascular phenotype

**Figure 5:**
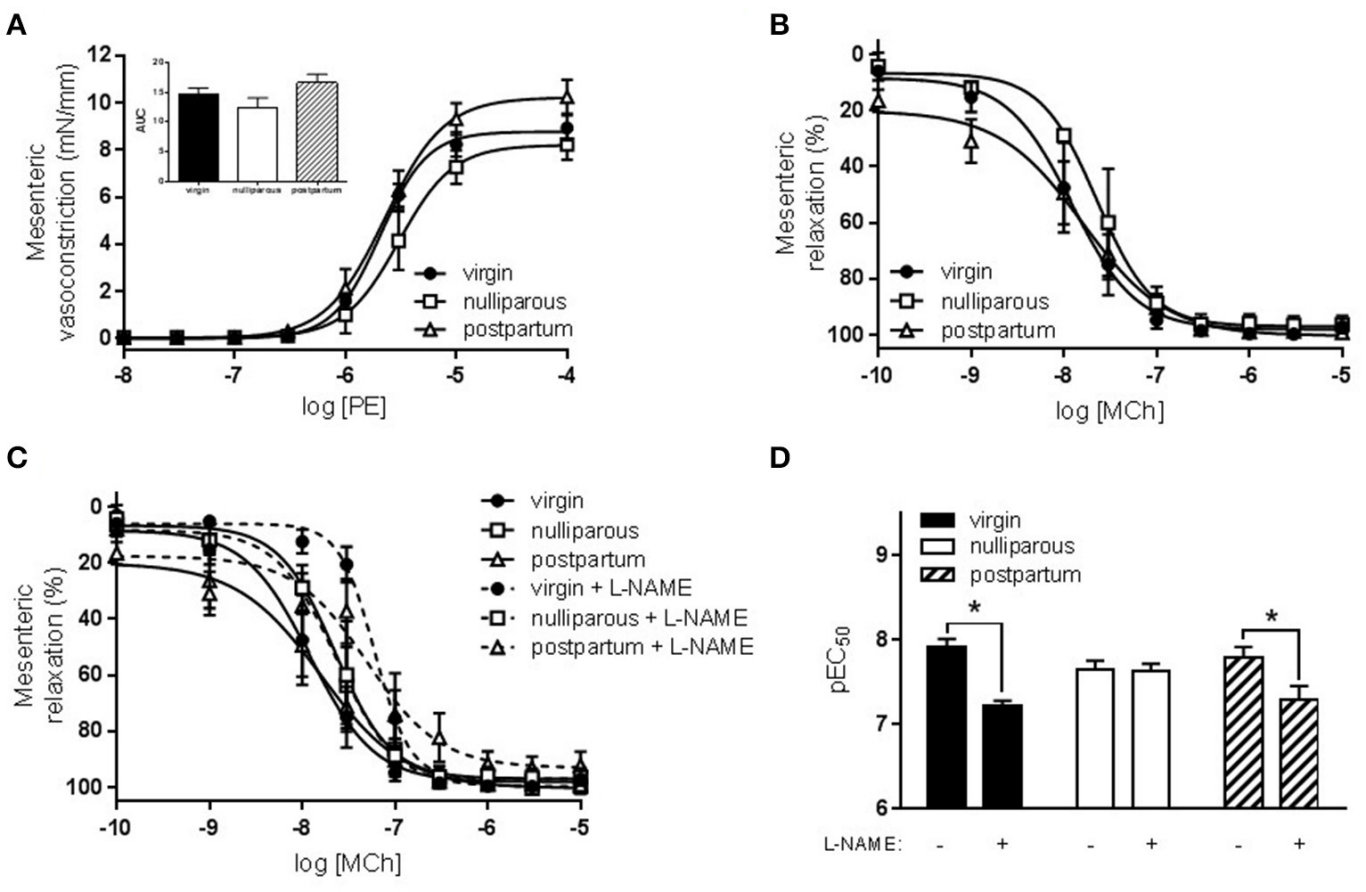
Constriction and relaxation responses of *mesenteric* arteries from advanced maternal age nulliparous or postpartum rats at 3 months postpartum, or equivalent age in never pregnant (virgin) controls (~13.5 months old). **(A)** Constriction to phenylephrine (PE, *n* = 5–11) and **(B)** relaxation to methylcholine (MCh, *n* = 4–13) were unaltered by maternal group. Inset shows one-way ANOVA analysis of area under curve (AUC) summary data. **(C)** Sensitivity to MCh was reduced in mesenteric arteries following inhibition of nitric oxide synthase (L-NAME). **(D)** Analysis of pEC_50_ summary data showed that L-NAME significantly reduced sensitivity to MCh-induced relaxation from virgin and postpartum, but not nulliparous, rats. The effect of parity and L-NAME on vasodilation to MCh (pEC_50_) were analyzed by two-way ANOVA: drug effect *p* < 0.01, with Sidak's post-test, ^*^p < 0.05.

In the mesenteric arteries, a comparison of all three groups (virgin, nulliparous, and postpartum) did not reveal the same pathological phenotype that was observed in the aorta. Responses to both KCl (data not shown) and PE (Figure 5A) were unaltered. PE responses in all three groups were also similarly increased by L-NAME (delta AUC: virgin 4.9 ± 1.3; nulliparous 7.4 ± 1.8; postpartum 4.9 ± 1.6; ns). Further, relaxation responses to MCh were unaltered between the three groups (Figure 5B). In the presence of L-NAME, however, sensitivity to MCh was significantly reduced in the virgin and postpartum groups but not in the nulliparous group, suggesting reduced NO modulation of vasodilation in rats which did not deliver (Figures 5C,D).

**Figure 6:**
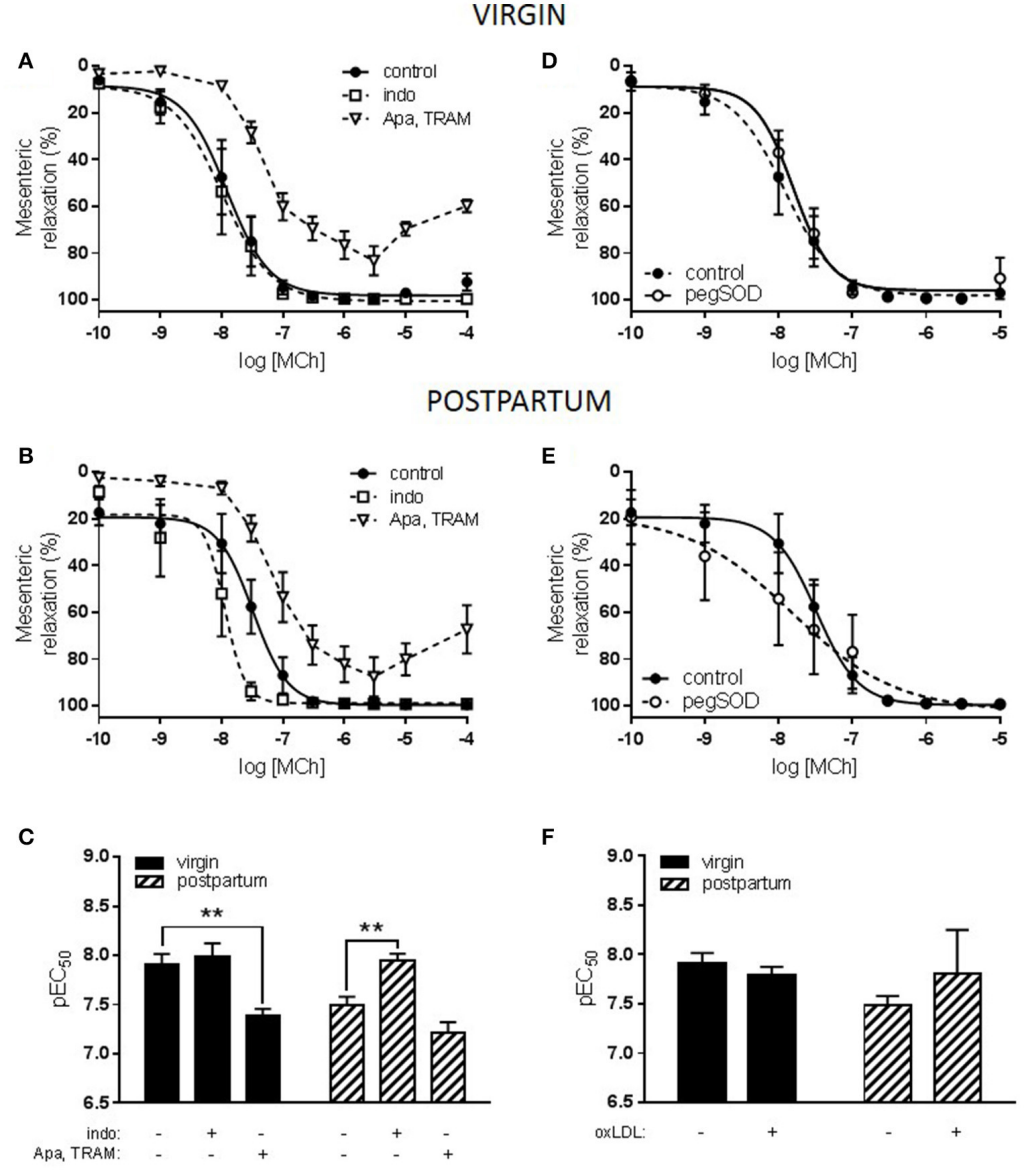
Effect of prostaglandin (PG) and endothelium-derived hyperpolarization (EDH) inhibition or superoxide scavenging on responses to methylcholine (MCh) in *mesenteric* arteries from advanced maternal age postpartum rats at 3 months postpartum, or equivalent age in never pregnant (virgin) controls (~13.5 months old). **(A)** Relaxation of mesenteric arteries from virgin rats to MCh was reduced by Apamin and TRAM-34 (Apa, TRAM; EDH inhibitors) but was unaffected by indomethacin (indo; PG inhibitor). **(B)** In postpartum rats, inhibition of MCh-induced relaxation by Apa, TRAM was reduced compared to the virgin control incubation while indo significantly enhanced MCh-induced relaxation. **(C)** The effect of parity and Apa, TRAM on vasodilation to MCh (pEC_50_) were analyzed by two-way ANOVA: drug effect *p* < 0.0001, with Sidak's post-test, ^**^p < 0.01, *n* = 5–6. Concurrent incubation with pegSOD (superoxide scavenger) did not affect MCh-induced vasodilation in either **(D)** virgin or **(E)** postpartum rats; however, the variability of MCh-induced relaxation was increased, *p* < 0.001 by Bartlett's test, in the presence of pegSOD in mesenteric arteries from postpartum rats. **(F)** The effect of parity and oxLDL on vasodilation to MCh (pEC_50_) were analyzed by two-way ANOVA with Sidak's post-test, *n* = 5-6.

Further investigation revealed that the contribution of endothelium-derived hyperpolarization (EDH) to MCh-induced relaxation in mesenteric arteries was reduced in the postpartum group compared to the virgin group (Figures 6A–C). In addition, a vasoconstrictive prostaglandin factor was evident in the postpartum group that was not present in the virgin group. MCh-induced relaxation was not affected by inclusion of a superoxide scavenger in either the virgin or postpartum groups (Figures 6D–F). In the presence of pegSOD, however, postpartum mesenteric artery responses to MCh demonstrated a significantly increased variability; Bartlett's test *p* < 0.001.

### Implantation failure

**Figure 7:**
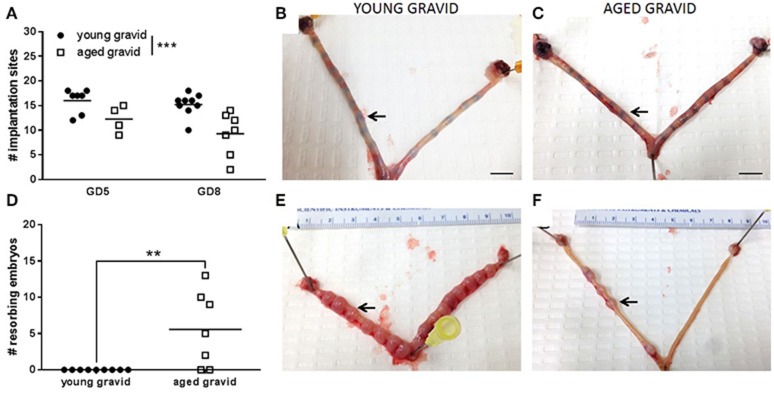
Identification of implantation sites and fetal viability in young (3–4 months old) and aged (9.5–10 months old) pregnancies. **(A)** At GD5 and GD8, aged rats had a reduced number of implantation sites. The effect of gestational age and maternal age on number of implantation sites were analyzed by two-way ANOVA: maternal age effect *p* < 0.001, *n* = 5–7. Representative images of GD5 uteri from **(B)** young and **(C)** aged rats with an implantation site identified by an arrow. **(D)** At GD8, the number of resorbing embryos were significantly increased (*p* < 0.01) in aged rats compared to young rats. Data were analyzed by *t*-test: *n* = 7–9. Representative images of GD8 uteri from **(E)** young and **(F)** aged rats with an implantation site identified by an arrow.

The number of implantation sites (at GD5 and GD8) and resorbing fetuses (at GD8; a point at which developing embryos could be identified) was investigated to determine the timing of litter loss in aging rats. 87.5% (7/8) of young dams carried a viable pregnancy (at least one viable implantation site) on GD5, compared to 66.7% (4/6) aged dams (*P* = 0.54, Fisher's exact test). 100% (9/9) of young rats carried a viable pregnancy on GD8, compared to 63.6% (7/11) aged dams (ns, Fisher's exact test). Uteri from rats which had zero implantation sites at either GD5 or GD8 were excluded from analysis since it could not be determined if these animals were gravid prior to this point. The number of implantation sites was slightly, but significantly, reduced at GD5 and GD8 in aged rats vs. young rats (Figures 7A–C). Correspondingly, the number of resorbing embryos was increased at GD8 in aged vs. young rats (Figures 7D–F); indicating that substantial loss occurs between GD5 and GD8.

## Discussion

The primary undertaking of the current study was to investigate the postpartum vascular phenotype in rats which became pregnant at an advanced maternal age, compared to those who were aged but not pregnant, to determine potential mechanisms of increased cardiovascular risk in later life. We demonstrated alterations in vascular endothelial function that may predispose rats that delivered at an advanced maternal age to cardiovascular disease. This study lends support to the epidemiological data which have shown increased post-partum systolic blood pressure in women who gave birth after the age of 35 years (Alonzo, 2002). Further, we demonstrated that pregnancy loss at an advanced maternal age may be indicative of a worsened vascular phenotype present at the time of gestation.

The investigation of maternal age and postpartum cardiovascular disease risk raises the issue of several confounding factors: parity, gravidity, age and potential additional age-related risk factors such as obesity. Due to these multiple complications, we assessed the relative risk of vascular dysfunction in animals whose only difference was a single pregnancy at an advanced maternal age. Age-matched “control” rats that were mated at a younger age were not used since these would have a postpartum period that would be considerably longer. Additionally, since there are epidemiological studies that suggest that pregnancy at a younger age is actually cardioprotective vs. nulliparity (Jacobs et al., 2012), a younger pregnant control would further complicate the comparisons.

A comparison of all three experimental groups (virgin, nulliparous and postpartum), demonstrated that the greatest pathological changes occurred in the nulliparous group; those which tested positive for sperm following mating but which did not deliver. Nulliparous rats presented an interesting group in that their vascular phenotype was worse than their postpartum counterparts which successfully delivered. Nulliparous rats demonstrated reduced aortic cholinergic (MCh)-mediated relaxation and a tendency to increased adrenergic (PE)-mediated constriction. Both aortic and mesenteric arteries demonstrated endothelial dysfunction which was due to a reduction in both basal and agonist-induced NO modulation. These alterations, which may result in a hyper-constrictive phenotype, could predispose nulliparous rats to cardiovascular diseases such as hypertension. The mesenteric arcade in particular is a critical component in the acute regulation of blood pressure given its ability to accommodate approximately 25% of total blood volume (Greenway, 1983; Rothe, 1983). Since the rats were all aged to over 13 months at the time of vascular assessment, aging would be expected to contribute to vascular dysfunction equally in all of the groups. However, in this study, pregnancy at an advanced maternal age may have acted as a “stress test” which uncovered a subgroup of rats that lost their pregnancy and may have had a worsened aging phenotype.

In order to further understand the nulliparous phenotype, the timing of pregnancy loss in aging rats was investigated in a subset of animals at GD5 and GD8. At GD5, the number of implantation sites was significantly reduced in aged rats compared to young pregnant rats. At GD8, a point at which the developing embryos could be identified, the number of implantation sites was reduced and the number of embryos being resorbed was increased; in line with human data which shows that the number of miscarriages is increased in women of advanced maternal age (Cleary-Goldman et al., 2005). In women with idiopathic recurrent miscarriage, polymorphisms of the genes encoding eNOS, resulting in lower plasma NO levels, and VEGF were found to be increased (Su et al., 2011). Further, altered NO production has been implicated in remodeling of the uterine endometrium and spiral arteries in pregnancy and, therefore, in early pregnancy loss (Najafi et al., 2012; Banerjee et al., 2013). These data, along with our results demonstrating altered basal and agonist mediated NO production in nulliparous rats, suggest that a pre- or ante-partum alteration in NO-mediated vascular pathways may be partially responsible for pregnancy loss. Miscarriage itself is associated with an increased risk of future cardiovascular disease (Parker et al., 2014); further implicating an underlying vascular dysfunction phenotype. However, the relative risk of a miscarriage vs. a successful pregnancy at an advanced maternal age has not been determined. Our data suggests that the underlying vascular pathology is worsened in rats which have lost their pregnancy. As it is not possible to predict which rats will lose their pregnancy, vascular function cannot be investigated in these animals pre-pregnancy. It might be inferred that this subset represents animals in which aging-related vascular dysfunction predisposed pregnancy loss.

While overall constriction and relaxation responses were preserved in the aorta, incubation of with oxLDL resulted in reduced sensitivity to MCh-induced relaxation in both virgin and postpartum rats. Since aging itself is associated with progressive vascular dysfunction (reviewed in Moreau and Hildreth, 2014; Camici et al., 2015), there may be an upregulation of scavenger receptors in both groups due to aging. In vessels from postpartum rats, our results demonstrated that there was a modest but significantly increased activation of LOX-1 receptors in response to oxLDL compared to aged virgin controls. The LOX-1 (reviewed in Ogura et al., 2009; Navarra et al., 2010) and PAF (reviewed in Shukla, 1992; Honda et al., 2002) receptors can have opposing effects on relaxation, activation of LOX-1 reduces NO-mediated relaxation while activation of PAF can increase PG-mediated relaxation, therefore, the presence of PAF receptor inhibitors can “unmask” a greater effect of oxLDL on the LOX-1 receptor; however, this effect was not seen in the current study. A downstream effect of LOX-1 receptor activation is increased superoxide production; however, oxLDL-mediated vascular dysfunction was not ameliorated by SOD in either the virgin or postpartum groups. The LOX-1 receptor has been linked to the pathogenesis of atherosclerosis (Mehta et al., 2006) and stroke (Yokota et al., 2016), therefore, increased activity of the LOX-1 receptor may be a key link to increased cardiovascular risk in the postpartum period following an advanced maternal pregnancy.

The vascular phenotype was further impacted in the mesenteric arteries of postpartum rats which, while they demonstrated maintained constriction and relaxation capacity, had an underlying shift in relaxation pathways. Of the three main endothelium-dependent pathways, NO was maintained, EDH reduced and a vasoconstrictor prostaglandin effect uncovered. A reduction of EDH mediated vasodilation is in-line with our data in the mesenteric arteries of advanced maternal age rats at GD20 (Care et al., 2015). This suggests that a change occurs in the vasculature in response to pregnancy that persists into the postpartum period and may lead to a predisposition to cardiovascular dysfunction; even following a successful pregnancy. While the presence of a superoxide scavenger did not significantly improve mesenteric artery relaxation, data in the presence of pegSOD was much more variable in the postpartum group compared to virgin controls; inviting speculation that some degree of oxidative stress was evident in this vascular bed.

In conclusion, we demonstrated aortic and mesenteric artery vascular dysfunction in postpartum rats following pregnancy at an advanced maternal age compared to age-matched, never-pregnant controls. Serendipitously, the inclusion of animals which were mated but never delivered in our studies uncovered a distinct vascular pathology in this group which could explain their early pregnancy loss. These data demonstrate mechanistic pathways that are altered and may lead to worsened outcomes in later life cardiovascular health and following a successful pregnancy at an advanced maternal age. Given the increasing prevalence of women delaying pregnancy, further investigation of this field is important in determining adequate care and management of this at-risk population.

## Author contributions

All experiments were performed in the laboratory of SD at the University of Alberta. JM data acquisition, analysis, drafted and edited manuscript AC conceptualization, data acquisition, methodology, critical revision of manuscript RK methodology, data acquisition, critical revision of manuscript CC conceptualization, critical revision of manuscript SD conceptualization, funding acquisition, critical revision of manuscript. All authors fully qualify for authorship, have approved the final version of the manuscript and are accountable for all aspects of the work in ensuring that questions related to the accuracy or integrity of any part of the work are appropriately investigated and resolved.

### Conflict of interest statement

The authors declare that the research was conducted in the absence of any commercial or financial relationships that could be construed as a potential conflict of interest.

## Abbreviations

AngII: angiotensin II
AUC: area under the curve
COX: cyclooxygenase
EDH: endothelium-dependent hyperpolarization
ET-1: endothelin-1
GD: gestational day
IK_Ca_: intermediate-conductance, Ca^2+^-activated potassium channel
L-NAME: N^G^-nitro-L-arginine methyl ester
LOX-1: lectin-like oxidized lipoprotein-1 receptor
MCh: methylcholine
NO: nitric oxide
oxLDL: oxidized low-density lipoprotein
PE: phenylephrine
pegSOD: polyethylene glycol superoxide dismutase
PG: prostaglandin
PAF: platelet activating factor receptor
PSS: physiological salt solution
SK_Ca_: small-conductance, Ca^2+^-activated potassium channel
TRAM-34: 1-[(2-chlorophenyl) diphenylmethyl]-1H-pyrazole.
